# Ceramide metabolism and cardiovascular risk factors: insights into therapeutic strategies

**DOI:** 10.3389/fcvm.2025.1656113

**Published:** 2025-09-04

**Authors:** Shouyi Yang, Yongxin Wu

**Affiliations:** ^1^ Department of Medicine, Kunming University of Science and Technology, Kunming, China; ^2^ Department of Cardiology, The First People’s Hospital of Yunnan Province, Kunming, China

**Keywords:** cardiovascular risk factors, ceramides, hypertension, diabetes mellitus, dyslipidemia, chronic kidney disease

## Abstract

Ceramides are components of sphingolipid metabolism and have been linked to cardiovascular disease (CVD) risk factors, including hypertension, insulin resistance, dyslipidemia and chronic kidney disease. Ceramide expression has been extensively studied but individual variability, species differences and regulatory mechanisms characterizing underlying relationships remain unclear. The current review analyzes roles of ceramides in the onset and progression of CVD and its risk factors. Diverse ceramide expression profiles are highlighted and recommendations for standardization of ceramide studies given. Ceramides are multifunctional and contribute to organ damage through dysregulated homeostasis during pathological processes. The current work emphasizes the importance of detecting ceramide imbalance and accounting for individual differences as a novel approach to metabolic research. Such a focus may enhance CVD management and give insights into ceramide-related pathologies.

## 1 Introduction

Cardiovascular disease (CVD) remains a leading global cause of mortality, responsible for approximately 20.5 million deaths or one-third of total deaths annually (1). CVD prevalence and mortality are on the rise due to changing population age structure, dietary habits and other lifestyle factors (2) and represent a substantial impact on patient quality of life and burden on families and healthcare systems. CVD is thus a global public health concern, for which early identification and management of risk factors are essential for prevention. Hjermann et al. (1) introduced the concept of cardiovascular metabolic syndrome, linking CVD with metabolic disorders, such as obesity, fatty liver and diabetes. Metabolic diseases are now well-established as key risk factors for atherosclerotic cardiovascular disease (ASCVD) and worsen patient prognosis. The American Heart Association expanded this concept in 2023, identifying “cardiovascular-kidney-metabolic syndrome”, a holistic interpretation of the shared pathophysiology underlying CVD, kidney dysfunction and metabolic abnormalities. The detrimental impact of dysregulated metabolism and circulation gives a theoretical framework to advance metabolic CVD treatment.

Sphingolipids, including ceramides (Cer), were discovered by Thudicum in the 19th century and identified as highly hydrophobic lipids with regulatory functions in nutrient metabolism and intracellular signal transduction. Sphingolipids have now been linked to the assessment, prediction and prognosis of adverse cardiovascular events and Cer are considered independent risk factors for CVD with abnormal Cer metabolism strongly associated with increased incidence of cardiovascular events (3, 4). An observational study involving 920 participants found elevated Cer to correlate with greater likelihood of adverse cardiovascular events (5) and Cer reduction has been shown to improve metabolic cardiovascular disease risk factors, such as diabetes and hypertension (6). The Coronary Event Risk Test (CERT 1/2) demonstrated the superiority of Cer over conventional biomarkers, such as low-density lipoprotein cholesterol (LDL-C) and total cholesterol, in predicting major adverse cardiovascular events (7). A lipidomics study conducted by Hilvo and colleagues on three prospective coronary heart disease cohorts from Europe (WECAC, KAROLA) and Australia (LIPID) developed and validated a novel risk assessment score, CERT 2, which includes six lipid species in a rapid and reliable clinical test. CERT 2 may be used alongside pre-existing risk assessment models, such as SMART and TIMI, and shows good predictive performance in various patient populations (7).

A four-year follow-up study of 495 patients who underwent non-acute coronary angiography showed a high Cer score (CERT ≥10) to be associated with twofold increased risk of all-cause mortality compared with a low score (CERT ≤2) (8). Comparable results were obtained by Qing et al. with Chinese patients with coronary artery disease, supporting the view that Cer has potential as a biomarker for prevention, treatment and prognosis.

The current review addresses the growing burden presented by CVD and its risk factors by synthesizing mechanistic roles of Cer in hypertension, dyslipidemia/obesity, chronic kidney disease and diabetes mellitus, emphasizing contributions to lipotoxicity, inflammation and metabolic dysfunction. Therapeutic strategies targeting Cer metabolism are explored, including neutral sphingomyelinase inhibitors for hypertension, dietary DHA/EPA supplementation for dyslipidemia and the pharmacological approach of the anti-diabetic, metformin. The clinical utility of Cer risk scores, such as CERT 1/2, which enhance risk stratification for major adverse cardiovascular events and guide precision therapy development are examined. The aim was to construct a framework by which Cer-related pathologies may be understood and to advance CVD therapeutic strategies.

## 2 Ceramide metabolism

Cer are sphingolipids composed of long-chain sphingosine and saturated fatty acids attached to an amino group and are components of organelle and cell membranes. There are three synthetic pathways: *de novo* synthesis, sphingomyelin hydrolysis and salvage. *De novo* synthesis begins with the reaction of L-serine and palmitoyl-CoA, catalyzed by serine palmitoyl transferase (SPT), followed by reduction to dihydrosphingosine, catalyzed by Cer synthecerase (CerS) and condensation to generate a 3-ketotic dihydrosphingosine. The action of dihydroceramide desaturase then produces Cer (9). The sphingomyelin hydrolysis and salvage pathways involve hydrolysis by resolvase enzymes, such as the neutral sphingomyyelase (NSMase) in the cell membrane and the acid sphingomyyelase (ASMase), to produce intracellular Cer (10). Sphingosine and sphingosine 1-phosphate (S1P) are formed by ceramidase and ceramide kinase (9, 10). Cer is involved in the metabolism of many organs when synthesized primarily in the endoplasmic reticulum and degraded to maintain a dynamic sphingolipid balance (11). High Cer promotes inflammation, dyslipidemia, diabetes, atherosclerosis and vascular endothelial dysfunction and represent risk factors for CVD.

**Figure 1:**
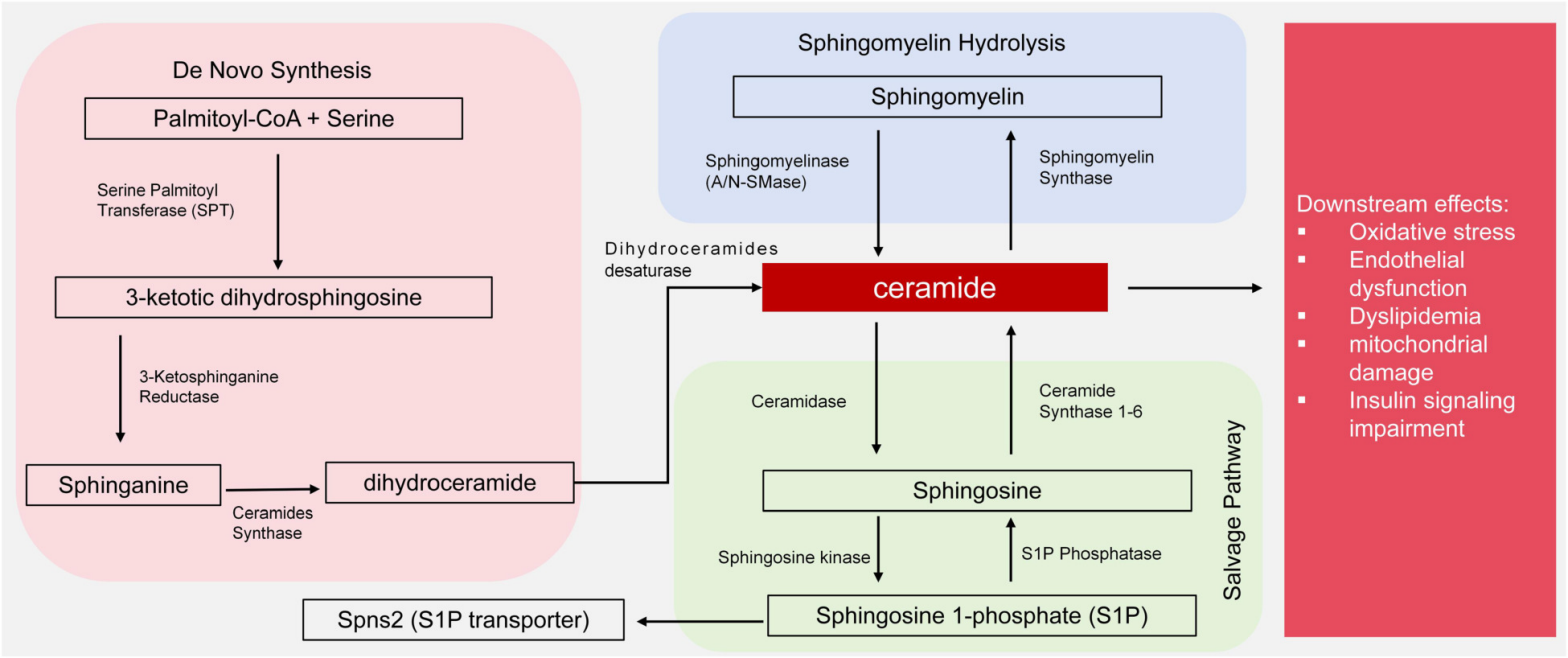
Ceramide synthesis by *de novo*, hydrolysis and salvage pathways and downstream effects of ceramide. *de novo* synthesis begins with palmitoyl-CoA and serine, catalyzed by serine palmitoyltransferase (SPT), leading to 3-ketodihydrosphingosine, dihydroceramide, and ceramide via subsequent enzymatic steps (reductase and dihydroceramide desaturase). Sphingomyelin hydrolysis, mediated by neutral sphingomyelinase (N-SMase) or acid sphingomyelinase (A-SMase), generates ceramide from sphingomyelin. The salvage pathway involves ceramidase converting ceramide to sphingosine, which can be further phosphorylated to sphingosine-1-phosphate (S1P) by ceramide kinase. Downstream effects of ceramide accumulation include oxidative stress, endothelial dysfunction, dyslipidemia, mitochondrial damage, and insulin signaling impairment.

As shown in Figure 1, dysregulation includes enhanced *de novo* synthesis via SPT or increased sphingomyelin hydrolysis by NSMase/ASMase and may cause Cer accumulation. Overactivation of these pathways contributes to oxidative stress and endothelial dysfunction in hypertension, lipid imbalances in dyslipidemia, mitochondrial damage in chronic kidney disease and insulin signaling impairment in diabetes (Figure 1). The use of SPT inhibitors or ceramidase modulators may thus have therapeutic potential.

## 3 Ceramides and cardiovascular risk factors

### 3.1 Ceramides and hypertension

#### 3.1.1 Evidence of association

The etiology of hypertension is multifactorial, involving oxidative stress, vascular endothelial dysfunction and reduced mitochondrial function, and incidence has risen steadily with time (2). Antihypertensive drugs achieve control of blood pressure in only 30% patients, highlighting the limitations of traditional treatments in addressing complex molecular pathologies (12). Novel therapeutic targets are required to improve management outcomes.

Cer have been linked to hypertension by numerous studies and circulating Cer have been suggested as biomarkers for conditions such as isolated nocturnal hypertension (13). A positive correlation was observed among Cer levels, NSMase activity and blood pressure in ovariectomized rat models (14, 15). High Cer often arises due to increased sphingomyelin hydrolysis (via NSMase), leading to a cascade of oxidative stress and vascular dysfunction. The reduction of blood pressure with angiotensin II receptor antagonists, such as losartan, or vasodilators has decreased Cer levels in clinical studies. Similarly, Cer synthesis inhibitors have been shown to lower blood pressure (16). Patients with hypertension often have endothelial dysfunction, sphingolipid dysregulation and elevated oxidative stress (17).

#### 3.1.2 Proposed mechanisms

Abnormal Cer production contributes to sustained vasoconstriction and increased oxidative stress, exacerbating endothelial dysfunction and elevating blood pressure (17–19). Cer promotes the conversion of nitric oxide (NO) into hydrogen peroxide (H_2_O_2_), increasing levels of reactive oxygen species (ROS), giving a self-reinforcing cycle of oxidative stress which stimulates Cer production, leading to endothelial dysfunction. NSMase inhibitors have been shown to reverse these deleterious effects (16, 20).

**Figure 2:**
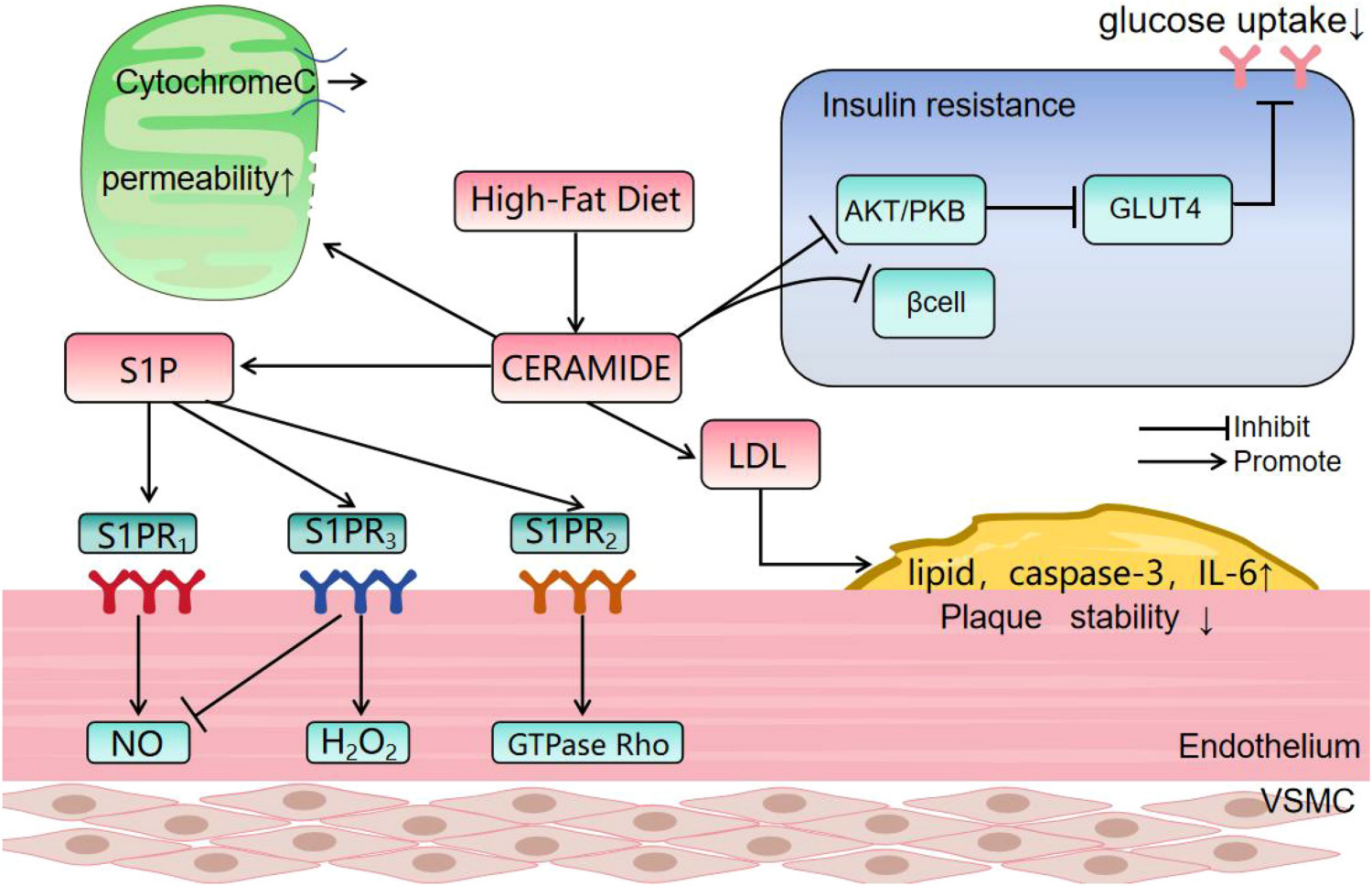
The effect of ceramide. PKB: Protein kinase B; GLUT4: Glucose transporter 4; S1PR: sphingosine −1-phosphate receptor; VSMC: Vascular Smooth Muscle Cells. (1) Ceramide causes dephosphorylation and inactivation of protein kinase B (Akt) by stimulating the activity of protein phosphatase A2. The inactivation of Akt prevents translocation of GLUT4 to the plasma membrane, reducing glucose uptake. (2) Ceramide forms large, stable channels in the outer mitochondrial membrane, increasing permeability and facilitating release of respiratory factor, cytochrome c. CerS6-derived ceramide interacts with mitochondrial fission factors, initiating fission and breakage. (3) Ceramide promotes LDL aggregation and plaque formation, and levels show an inverse relationship with vascular smooth muscle cell numbers which normally stabilize atherosclerotic plaques. Cer levels correlate positively with plaque lipid and caspase-3 and with inflammatory cytokines, such as IL-6. (4) Binding of S1P to S1PR1 enhances NO production and binding to S1PR2 may inhibit this effect and promote H_2_O_2_ production. Binding to S1PR3 activates the GTPase, Rho, and stimulates inflammatory responses.

Spinster homolog 2 (Spns2) is an S1P transporter which is involved in lipid signaling cascades. Spns2 deficiency leads to Cer accumulation, impaired endothelial signal transduction, reduced respiratory capacity and mitochondrial dysfunction. Apoptosis is triggered and impaired vasodilation contributes to early-stage cardiovascular disease. Inhibition of *de novo* Cer synthesis has been found to improve mitochondrial function (21, 22) and S1P modulates the balance between NO and H_2_O_2_ with an impact on blood pressure regulation. These effects are dependent on the expression of S1PR receptor subtypes. Activation of endothelial S1PR1 promotes NO production, leading to vasodilation (23) but activation of S1PR2 and S1PR3 by Cer-mediated S1P signaling triggers stress fiber formation and adhesion junction disassembly via GTPase Rho activation in endothelial cells (Figure 2). This cascade increases endothelial permeability and activates the p38 SAPK and NF-*κ*B pathways, contributing to vascular remodeling and inflammation. In addition, S1PR3 activation induces H_2_O_2_ production, promoting vascular smooth muscle contraction and vasoconstriction (23, 24).

#### 3.1.3 Findings from intervention studies

Clinical studies have correlated decreased blood pressure with reduced Cer levels. Angiotensin II receptor antagonists, such as losartan, vasodilators or Cer synthesis inhibitors achieve the reduction of circulating Cer and of blood pressure (16). The Cer pathway has also been suggested to reduce blood pressure via smooth muscle relaxation and vasodilation (18), although precise mechanisms remain unclear.

#### 3.1.4 Potential therapeutic strategies

In summary, abnormal Cer synthesis has been associated with hypertension and vascular disease. Enzymes and receptor subtypes involved in Cer metabolism contribute to increased blood pressure via effects on oxidative stress and endothelial dysfunction. Inhibition of such pathways may reduce blood pressure and improve vascular endothelial function and further research may allow the development of innovative anti-hypertension therapeutic strategies.

### 3.2 Ceramides and dyslipidemia/obesity

#### 3.2.1 Evidence of association

ASCVD accounted for 61% of China's CVD burden in 2019 and elevated LDL-C is the second-largest ASCVD risk factor (25). Statins are the primary treatment for dyslipidemia and ASCVD but improved therapeutic strategies are required to tackle rising prevalence and its increasing incidence in younger populations (2). Dyslipidemia describes a pathological imbalance in circulating lipids, characterized by elevated LDL-C and reduced high-density lipoprotein cholesterol (HDL-C). The imbalance often stems from increased fat intake and metabolic dysfunction, particularly the accumulation of saturated fatty acids in skeletal muscle and liver. Such imbalances promote insulin resistance, inflammation and increased Cer production (26).

Hyperlipidemia and elevated Cer are frequently concomitant. Saturated fatty acids stimulate Cer accumulation through upregulation of *de novo* synthesis and the sphingomyelin pathway (27, 28). Species-specific accumulation of Cer occurs, with Cer16:0, Cer18:0 and Cer24:1 increasing while Cer24:0 decreases, disrupting lipid homeostasis and promoting atherosclerosis (28, 29). Cer24:0 levels have been positively correlated with LDL-C in elderly patients with coronary heart disease and diabetes (30), perhaps reflecting disease-specific or genetic predispositions.

#### 3.2.2 Proposed mechanisms

The liver is a central organ in lipid metabolism and the primary source of plasma Cer. Increased fatty acids may stimulate liver Cer synthesis and secretion, rather than increasing SPT activity, to protect against the harmful effects of intracellular Cer accumulation (31). Hepatocyte Cer promotes fatty acid uptake by an impact on the subcellular distribution of CD36 and induces triglyceride synthesis and lipid metabolism via an effect on Srebf1 expression. These effects mitigate dyslipidemia (32). However, excessive Cer production induces oxidative stress (18), mitochondrial dysfunction (22) and impaired respiratory function (21). These observations support the notion that plasma Cer correlate with liver fat content rather than causing liver dysfunction (33).

#### 3.2.3 Findings from intervention studies

Fatty acids are precursors for sphingolipid synthesis and excessive intake results in elevated Cer. Experiments with high-fat diets have demonstrated that krill oil and DHA/EPA supplementation lower liver Cer16:0 and serum Cer18:0, Cer22:0 and Cer24:0 while increasing liver Cer22:0 and Cer24:0 (29). Such supplements may ameliorate metabolic disorders caused by high-fat diets by an impact on Cer metabolism. Oxidized low-density lipoprotein (oxLDL) may be taken up by macrophages in the vascular endothelium, forming foam cells which contribute to lipid stripes and fibrous plaques during atherosclerotic progression. Cer is inversely proportional to the number of vascular smooth muscle cells and exacerbate plaque instability (34). Cer also correlates positively with the pro-inflammatory molecules, IL-6 and MCP-1, promoting plaque lipid and caspase-3 (35). The ASMase/Cer pathway also facilitates lipoprotein aggregation and foam cell formation, accelerating atherosclerosis (34). Cer have been incorporated into predictive tools, such as the CERT 2 score, and outperform the traditional LDL-C biomarker in forecasting major adverse cardiovascular events in patients with coronary artery disease, illustrating clinical relevance in dyslipidemia (7). Cer profiling has the potential to inform targeted interventions for dyslipidemia and atherosclerosis.

HDL is protective, unlike LDL-C, and lowers Cer levels by promoting the release the anti-inflammatory and antioxidant NO. HDL binds scavenger receptor class B type I (SR-BI) and affects Cer by a pathway that is independent of calcium phosphorylation or Akt kinase activation. Endothelial NO synthase is stimulated and NO released to mitigate atherosclerosis (36). Cer thus have a pleiotropic role in lipid metabolism with effects dependent on interactions with upstream regulators, such as LDL and HDL.

#### 3.2.4 Potential therapeutic strategies

The targeting of Cer synthesis has potential for dyslipidemia treatment. The fragransin inhibitor of *de novo* Cer synthesis reduced hepatic steatosis, lobular inflammation and ballooning and improved dyslipidemia (37). The combination of nicotinamide ribose and pterostilbene enhanced liver function and lowered circulating Cer14:0 levels compared with a placebo (38). These findings suggest that regulation of Cer synthesis may slow the progression of dyslipidemia to atherosclerosis and alleviate pathological manifestations, giving additional therapeutic options for dyslipidemia patients.

### 3.3 Ceramides and chronic kidney disease

#### 3.3.1 Evidence of association

Chronic kidney disease (CKD) is characterized by the progressive impairment of renal function, manifesting as persistent urinary abnormalities, structural kidney damage or impaired excretory function. The global prevalence of CKD was 18.99 million in 2019, resulting in 41.54 million disability-adjusted life years (DALYs) (39). The economic burden associated with dialysis and other nephropathy treatments places substantial pressure on patients and healthcare systems and CKD prevention and mitigation remain the focus of research.

Nephropathy patients have dysregulated circulating Cer (40). Cer is abundant in the kidney and have been implicated in the pathogenesis of ischemia-reperfusion, toxic and acute kidney injuries driven by oxidative stress (41). CKD is a dynamic and multifaceted pathological process and renal tubular epithelial cells of the kidney parenchyma are involved in disease progression (42).

#### 3.3.2 Proposed mechanisms

Excessive Cer affected glomerular filtration and promoted epithelial cell death in the renal proximal convoluted tubule in animal experiments (43). *De novo* synthesis may be upregulated or salvage pathways impaired in CKD, compromising mitochondrial integrity and accelerating renal fibrosis. Indeed, renal tubular atrophy and basement membrane thickening are frequently observed in the renal cortices of diabetic mice. Protein droplets, collagen deposition and increased expression of CerS6 mRNA, Cer (d18:1/14:0) and Cer (d18:1/16:0) are also present (44). Excessive fatty acid accumulation in the kidney impairs tubular uptake and fatty acid oxidation and Cer activate fatty acid esterification and storage to mitigate this effect (47). However, increased Cer are detrimental to mitochondrial function, forming large, stable channels in the outer membrane which affect the membrane platform and increase permeability, facilitating the release of cytochrome C (41). Cer also affects electron transport chain activity and those derived from CerS6 initiate mitochondrial fission and fragmentation (23).

#### 3.3.3 Findings from intervention studies

NanoZnO treatment has been linked to increased death of renal juxtamedullary convoluted tubule epithelial cells due to elevated intracellular Cer. However, the ceramide synthetase inhibitor, fumarisin, reduced CerS6, reduced renal tubulointerstitial fibrosis and prolonged survival of diabetic mice (45). Cer24:0 has been suggested to protect against endoplasmic reticulum stress, consistent with findings that reduced Cer24:0 is associated with renal fibrosis (40, 46). By contrast, high levels of Cer16:0, Cer18:0 and Cer24:1 have been linked to a rapid decline in renal function (40).

#### 3.3.4 Potential therapeutic strategies

Elevated Cer levels induced programmed cell death and fibrosis in damaged kidney, perhaps as an adaptation to prevent uncontrolled cell lysis. Harmful effects of Cer on the kidney are mediated through mitochondrial dysfunction, vascular endothelial dysfunction and excessive programmed cell death.

### 3.4 Ceramides and diabetes mellitus

#### 3.4.1 Evidence of association

Diabetes mellitus is characterized by chronic hyperglycemia and microangiopathy and progression is linked to disturbed glucose and lipid metabolism. Diabetic insulin resistance and β-cell dysfunction increase the risk of CVD in addition to an impact on glycemic control. Data from the China National Hospital Quality Monitoring System show a 21% incidence of microvascular and 18.4% incidence of macrovascular lesions in patients with type 2 diabetes (T2D) (48). Thus, mitigation of insulin resistance is central to both diabetes management and reduction of CVD risk. Elevated plasma Cer16:0, Cer18:0, Cer20:0 and Cer22:0 correlate with higher HOMA-IR values, β-cell damage and an increased T2D risk. Lipidomic studies show increased Cer in T2D but decreased levels in type 1 diabetes (T1D), highlighting differences in disease mechanisms associated with risk (49–51).

#### 3.4.2 Proposed mechanisms

The inhibition of insulin signaling by Cer leads to reduced skeletal muscle glucose uptake and increased hepatic gluconeogenesis and glycogenolysis, impairing blood glucose regulation. Cer activates protein phosphatase A2 and inhibit the Akt/PKB phosphorylation necessary for insulin signaling. GLUT4 expression is reduced and β-cell apoptosis induced which reduces insulin secretion (52, 53). Cer accumulates due to increased *de novo* synthesis from saturated fatty acids or reduced breakdown by ceramidase, perpetuating insulin resistance and β-cell dysfunction.

Abnormal Cer accumulates and impairs mitochondrial function, causing mitochondrial DNA damage (54) and lowering respiratory activity (21). A self-perpetuating cycle of Cer accumulation and metabolic dysfunction results. Plasma Cer increase significantly with excessive consumption of saturated fats, causing insulin resistance but not necessarily weight gain (55). The obesity which is often associated with dyslipidemia is another contributory factor and lipid accumulation facilitates the development of insulin resistance and T2D. Lipoproteins devoid of Cer fail to induce insulin resistance in muscle cells but approximately 98% circulating Cer are normally bound to lipoprotein subcomponents, evenly distributed in LDL and HDL cholesterol (56). Thus, Cer link saturated fatty acids to the inhibition of insulin signaling, indicating a significant role in metabolic regulation.

#### 3.4.3 Findings from intervention studies

Genetically engineered C2C12 myocytes that converted Cer to sphingosine were used to demonstrate that acid ceramidase overexpression both prevented the accumulation of Cer and reduced the inhibition of insulin signaling due to saturated fatty acids (57). Exercise, metformin, pioglitazone and dietary modifications have been shown to enhance insulin sensitivity and reduce Cer levels (58). In addition, knockdown of CerS6 by antisense oligonucleotides decreased Cer(d18:1/18:0) and Cer16:0, reduced fat and improved glucose tolerance and insulin sensitivity in a mouse model (59).

#### 3.4.4 Potential therapeutic strategies

Cer clearly play a role in diabetic pathophysiology, linking insulin resistance and CVD and mediating the inhibitory effects of saturated fatty acids on insulin signaling. Observed gender differences in Cer production may enable precision medicine tailored to individual patients.

### 3.5 Ceramides and cardiovascular events

#### 3.5.1 Evidence of association

A clinical causal relationship between altered Cer profile and cardiovascular events has been shown. A prospective European cohort study (*n* = 495) showed an elevated Cer risk score (CERT ≥ 10) to double all-cause mortality. Predictive efficacy exceeded that of pre-existing coronary calcium scoring, giving a novel tool for early risk stratification (8, 15). A 1 μmol/L increase in Cer24:0 correlated with LDL-C levels in Chinese patients with coronary artery disease and diabetes, suggesting vasoprotective properties in Asian populations (30). Therapy targeted to acid ceramidase reduced mortality post-myocardial infarction, a benefit attributed to the suppression of Cer20:0/Cer24:1, indicating the clinical value of precision therapy (69). Lower plasma Cer18:0 levels have been demonstrated in male T2D patients compared with females, emphasizing the utility of sex-stratified management of CVD (51).

#### 3.5.2 Proposed mechanisms

The axis by which Cer increase cardiovascular risk begins with the *de novo* synthesis (SPT/CerS-regulated) and sphingomyelin hydrolysis (NSMase/ASMase-mediated) promoted by saturated fatty acids which leads to an altered circulating profile (Cer16:0/Cer18:0↑, Cer24:0↓) that triggers mitochondrial respiratory chain dysfunction and oxidative stress (9, 22, 54). Cer accumulation within the vasculature leads to conversion of NO to H_2_O_2_, activation of the S1PR2/3-Rho axis and vascular smooth muscle contraction (16, 23). Hepatic Cer16:0 upregulates CD36 membrane translocation, promoting fatty acid uptake and oxidized LDL deposition (32, 34). Renal CerS6 derivatives form mitochondrial channels, releasing cytochrome c and inducing tubular epithelial apoptosis and fibrosis (44, 47). Cer inhibit Akt phosphorylation in pancreatic β-cells, disrupting insulin signaling and acting synergistically with mitochondrial DNA damage to exacerbate insulin resistance (53, 54). Increased inflammatory cytokines (IL-6/MMP-9↑), lipotoxicity and fibrotic cascades are stimulated by multi-tissue damage and promote atherosclerotic plaque rupture and cardiorenal metabolic syndrome (35, 42).

#### 3.5.3 Findings from intervention studies

The angiotensin II receptor antagonist, losartan, and NSMase inhibitors reduced circulating Cer and improved systolic pressure and endothelial function for hypertension management (16, 20). In addition, DHA/EPA supplementation decreased serum Cer18:0/Cer24:0 and increased hepatic Cer22:0 in a synergistic action with the fragransin-mediated inhibition of *de novo* lipid synthesis to ameliorate dyslipidemia (29, 37). Reduction of Cer(d18:1/18:0) by CerS6 antisense oligonucleotides blocked Akt phosphorylation and alleviated diabetic insulin resistance (59). Targeted acid ceramidase therapy also reduced post-MI mortality and Nogo-A-preserved mitophagy delayed progression to heart failure (69, 70).

#### 3.5.4 Potential therapeutic strategies

Therapies targeting Cer metabolism dysregulation might adopt an integrated approach of pathway modulation, subtype-specific targeting and individualized clinical application to reduce cardiovascular risk. Pharmacological inhibition of *de novo* synthesis and sphingomyelin hydrolysis may alleviate lipotoxicity, enhance insulin sensitivity and restore mitochondrial integrity. The concurrent stimulation of Cer degradation would facilitate conversion to the protective S1P, improving endothelial function.

The reduction of pathogenic short-chain Cer variants, Cer16:0, Cer18:0, combined with elevation of the cardioprotective long-chain, Cer24:0, is likely to optimize organ-specific outcomes. It has been shown that inhibition of CerS6 alleviated renal fibrosis and modulation of the Cer24:0/Cer24:1 ratio stabilizes atherosclerotic plaques.

#### 3.5.5 Individual ceramide differences

Differences in Cer levels due to racial, gender and age cause variability in CVD susceptibility and progression (Sections 2.1–2.4). Higher baseline Cer may amplify risks, such as dyslipidemia or diabetes, and influence responses to Cer synthesis inhibitors. The tailoring of CVD prevention and treatment must take account of these differences, emphasizing the significance of a personalized approach. Ethnic and gender-based differences have received little attention in previous studies of Cer levels but cross-sectional analyses by high-performance liquid chromatography tandem mass spectrometry (HPLC/MS-MS) have revealed racial variations. Total Cer was higher in African Americans compared with Caucasians but Cer16:0, Cer20:0, Cer24:0 and Cer24:1 were elevated in Caucasians with metabolic disorders (MetD) compared with African Americans (*p* < 0.05) (60). Chinese subjects had reported plasma levels of 0.28 μmol/L Cer16:0 (*p* < 0.01), 0.075 μmol/L Cer18:0 (*p* < 0.01), 10.785 μmol/L Cer24:0 (*p* < 0.03) and 3.435 μmol/L Cer24:1 (*p* < 0.01), all of which were higher than in African Americans or Caucasians (30).

A study of 71 healthy student volunteers found Asians to have the highest ceramide-to-cholesterol ratio with no significant differences among Cer subgroups (61). These findings suggest higher plasma Cer levels in Asians than in other groups but further research with larger sample sizes is needed to confirm and evaluate therapeutic implications.

Impacts of gender and age on Cer levels are less well understood. Plasma Cer(d18:1/24:0) and Cer(d18:1/24:1) have been observed to increase with age in women and plasma Cer(d18:1/24:1) correlated inversely with plasma estradiol levels across all female age groups (61). *In vitro* experiments with human estrogen receptor positive cancer cells have demonstrated that estradiol inhibited Cer biosynthesis and promoted degradation, reducing total Cer level (62).

Elevated Cer in muscle tissue was associated with increased risk of diabetes, higher body fat ratio and lower insulin sensitivity in men. By contrast, adipose tissue shows a more complex relationship with Cer in women, perhaps influenced by estrogen regulation. Changes in Cer metabolism linked to type 1 diabetes (T1D) appear more pronounced in males with lower Cer(d18:1/20:0) and Cer(d18:1/18:0) observed. In addition, 1-deoxyceramides (m18:1/20:0) were associated with type 2 diabetes (T2D) in women but not in men. Gender may thus be an independent factor influencing Cer metabolism and its role in diabetes (51).

A review of patients with coronary endothelial dysfunction (CFR > 2) between 1992 and 2019 produced contrary results and no relationship of plasma Cer with sex or correlation with age was found (63). However, substantial age and sex-related differences have been reported elsewhere. For instance, sphingolipid concentrations were lower in females than males at ages 18–39 but higher in females at ages 56–70 (64). A cohort study of 164 participants aged 19–80 showed a positive correlation of age with Cer(d18:1/24:0) (*p* = 0.0198) and Cer(d18:1/24:1) (*p* < 0.0001). Cer(d18:1/24:1) was positively associated with age in males (*p* = 0.0179) but negatively correlated with plasma estradiol in females (*p* = 0.007) (63).

## 4 Summary and outlook

The association between Cer and CVD is well-established and plasma Cer risk scores, such as CERT 1, have been used for clinical prediction (64). Cer is known to mediate lipotoxicity, contributing to inflammation, impaired insulin signaling and apoptosis, which promotes CVD risk factors, such as hypertension, diabetes, dyslipidemia and kidney disease (Table 1). Cer has an impact throughout CVD progression. A Mayo Clinic cohort study identified Cer score as a robust predictor of CVD risk stratification and major adverse cardiovascular events (65). Cer levels remained a statistically significant predictive marker even after clinical treatment. CERT 1/2 scores integrate Cer species, Cer16:0, Cer18:0 and Cer24:1, into a practical clinical tool to stratify CVD risk and monitor therapeutic responses, particularly in patients with dyslipidemia and diabetes. Elevated Cer levels inform the use of targeted interventions, such as inhibitors of ceramide synthesis or dietary modifications, to mitigate cardiovascular risk. Disruption of *de novo* synthesis, sphingomyelin hydrolysis and salvage mechanisms allow Cer to drive risk factors, underscoring the need for therapies that restore sphingolipid balance, including pathway-specific inhibitors and lifestyle interventions.

**Table 1:** Relationship between ceramide and cardiometabolic diseases.

Disease	Relationship with ceramide	Intervention	Outcomes	Ref.
Hypertension	↑ General ceramide elevation	Losartan (angiotensin II receptor blocker)	↓ Blood pressure and ceramide levels	(16)
	↑ NSMase activity	NSMase inhibitors	Improved endothelial function, reduced oxidative stress	(13–16, 20)
Dyslipidemia/Obesity	↑ Cer16:0, Cer18:0, Cer24:1; ↓ Cer24:0	Fragransin, nicotinamide ribose + pterostilbene, krill oil, DHA/EPA	↓ Hepatic steatosis, ↓ LDL-C, improved lipid profile, ↓ inflammation and plaque instability	(28, 29, 37)
Chronic Kidney Disease	↑ Cer16:0, Cer18:0, Cer24:1;	Fragransin	↓ Tubular fibrosis, ↓ epithelial cell death	(40, 44, 45)
Diabetes Mellitus (T2D)	↑ Cer16:0, Cer18:0, Cer20:0, Cer22:0	Exercise, metformin, pioglitazone, antisense oligonucleotides targeting CerS6	↓ Improved insulin sensitivity, ↓ HOMA-IR improved glucose tolerance	(57–59)
Myocardial infarction	↑ Cer16:0, Cer20:0, Cer20:1, Cer24:1	Modified mRNA [modRNA] therapy for Acid Ceramidase	↓ Ceramide levels ↓ cell death rates, ↓ proinflammatory detrimental neutrophils.	(69)
heart failure	↑ Cer16:0, Cer14:0, Very-long-chain ceramides	Nogo-A (a negative regulator of SPT activity)	Preserving beneficial autophagy, mitochondrial function, and metabolic gene expression limits the progression towards HF under sustained stress.	(70)

Future studies should prioritize the evaluation of population differences and the standardization of Cer measurement in different tissues to allow assessment of potential organ damage. Dietary fat intake should be carefully monitored during research. C22:0, C24:0 and C18:0 Cer have been negatively correlated with vegetable intake (*r* = −0.679, *p* < 0.05; *r* = −0.711, *p* < 0.05; *r* = −0.808, *p* < 0.01) and C24:0 with soy intake (*r* = −0.736, *p* < 0.05) (61, 66). Mechanisms causing racial and gender differences in Cer levels remain unclear. The integration of racial and gender variations in Cer profile into CVD management is essential, since increased susceptibility to diabetes and dyslipidemia in Asian males is an example that affects both risk and treatment response, including that to NSMase blockers or dietary interventions. Large-scale, diverse cohorts should be investigated to standardize Cer measurement, account for differences in CERT 1/2 and develop tailored therapies to optimize outcomes.

Cer represents promising therapeutic targets for reducing CVD risk, but limitations remain. Changes to downstream metabolites in the sphingolipid pathway may disrupt protective functions. For instance, lactosylceramides (LacCer) are glycosylated Cer species with an inverse association with insulin resistance and type 2 diabetes in human cohort studies. LacCer 14:0, 16:0 and 24:1 were linked to reduced diabetes risk, perhaps due to anti-inflammatory or insulin-sensitizing effects (67). Reduced Cer synthesis may lower beneficial LacCer species, exacerbating metabolic dysfunction or offsetting therapeutic gains in diabetes and dyslipidemia. In addition, S1P promotes vasodilation and endothelial protection via S1PR1 and may be reduced if Cer levels are lowered. Over-suppression may lead to the vasoconstriction, increased oxidative stress and mitochondrial dysfunction observed in animal models where LacCer accumulation contributed to diabetic mitochondrial impairment (68). Broader metabolic implications include potential off-target effects on lipid homeostasis, inflammation or apoptosis, particularly given species and individual differences in Cer metabolism. These potential risks highlight the need for selective inhibitors to target pathogenic Cer species, Cer16:0 and Cer18:0, without broader pathway effects and incorporation of CERT scores for monitoring of efficacy and safety. Future studies should prioritize randomized controlled trials to evaluate adverse effects, dose-response relationships and interactions with comorbidities to ensure net benefits for Cer modulation in CVD management.
